# Metagenomic data of fungal internal transcribed Spacer and 18S rRNA gene sequences from Lonar lake sediment, India

**DOI:** 10.1016/j.dib.2015.06.001

**Published:** 2015-06-18

**Authors:** Pravin Dudhagara, Anjana Ghelani, Sunil Bhavsar, Shreyas Bhatt

**Affiliations:** a Department of Biotechnology, Veer Narmad South Gujarat University, Surat 395007, India; b Department of Microbiology, Shree Ramkrishna Institute of Computer Education and Applied Science, Surat, India; c Department of Biosciences, Veer Narmad South Gujarat University, Surat, India; d Department of Life Sciences, Hemchandrachrya North Gujarat University, Patan, India

**Keywords:** Fungal ITS, 18S rRNA, Metagenome, Lonar Lake, fTEFAP

## Abstract

The data in this article contains the sequences of fungal Internal Transcribed Spacer (ITS) and 18S rRNA gene from a metagenome of Lonar soda lake, India. Sequences were amplified using fungal specific primers, which amplified the amplicon lined between the 18S and 28S rRNA genes. Data were obtained using Fungal tag-encoded FLX amplicon pyrosequencing (fTEFAP) technique and used to analyze fungal profile by the culture-independent method. Primary analysis using PlutoF 454 pipeline suggests the Lonar lake mycobiome contained the 29 different fungal species. The raw sequencing data used to perform this analysis along with FASTQ file are located in the NCBI Sequence Read Archive (SRA) under accession No. SRX889598 (http://www.ncbi.nlm.nih.gov/sra/SRX889598).

## 1 Specifications table

**Table:** 

Subject area	Microbiology, mycology, biodiversity
More specific subject area	Metagenomics
Type of data	Table
How data was acquired	Fungal tag-encoded FLX massively parallel pyrosequencing using Roche GS 454 FLX Titanium sequence followed by analysis using PlutoF 454 pipeline.
Data format	Raw data FASTQ file
Experimental factors	ITS region along with partial gene sequence of 18S rRNA were amplified using fungal specific primers (ITS1-F and ITS4-F) from isolated metagenome followed by pyrosequencing using GS 454 FLX Titanium chemistry.
Experimental features	The sediment sample was collected from 3 m deep from Lonar soda lake, India.
Data source location	Lonar Lake, Lonar city, India.
Data accessibility	Data are available at NCBI Biosample under accession No. SAMN02486448 and SRA accession No. SRX889598

## 2 Value of the data

• This data provides a comprehensive survey and quantitative picture of fungal diversity in Lonar lake.
• Data is applicable for the comparative study of the different Crater lakes to generate the fungal profile based on 18S rRNA and ITS sequences.
• Chances to detect the unculturable and novel fungal species in the lake metagenome.
• Data insights the abundance, diversity, distribution and coexisting of the fungi.
• Accessibility of raw sequencing data allows researchers to perform their secondary analysis using new tools.

Direct link to the data: https://www.ncbi.nlm.nih.gov/biosample/SAMN02486448

## 3 Data, experimental design, materials and methods

### 3.1 Sampling

Brownish Black sediment samples were collected at 3-m depth from the Lonar soda lake (19°97′67′′N, 76°50′83′′E), Maharashtra state, India. Temperature during sampling was reported 30 °C and pH was 9.8. Samples have been brought to the laboratory on the same day for the isolation of metagenomic DNA.

### 3.2 DNA extraction

Metagenomic DNA was isolated by the soil DNA isolation kit PowerMaX™ (MO BIO Laboratories, Inc., CA, USA). All the steps in the isolation procedure were carried out as per manufacturer instructions. Finally, the extracted metagenomic DNA was checked using 0.8% w/v agarose gel electrophoresis to verify the success of the extraction. Pooled DNA sample was quantified using a Nanodrop spectrophotometer (Nyxor Biotech, Paris, France).

### 3.3 Sequencing

To amplify the region of ITS, PCR and secondary PCR procedures were performed as described by Leake et al. [1]. The amplicon was greater than 500 bp that partially covers 18S rRNA gene and ITS region of the fungi. A pair of fungal specific primer ITS1-F 5′CTTGGTCATTTAGAGGAAGTAA and ITS4F 5׳ TCCTCCGCTTATTGATATGC were used to amplify the partial 18S rRNA with ITS regions and carry out the fTEFAP. An fTEFAP is a universal fungal identification method like bacterial tag-encoded FLX amplicon pyrosequencing (bTEFAP) [2,3]. These primers amplified ITS regions between the 18S and 28S rRNA genes with partial coverage of 18S rRNA sequence. Pyrosequencing was performed using GS 454 FLX instrument with Titanium reagents. The fTEFAP sequencing were based upon titanium protocols (Roche, Indianapolis, IN, USA) and procedures have been performed at the Research and Testing Laboratory (RTL) (Lubbock, TX, USA) based upon RTL protocols (www.researchandtesting.com) [4].

### 3.4 Data analysis

Output file containing ITS sequences with partial 18S rRNA gene sequence were analyzed using PlutoF 454 pipeline tool (Table 1) [5]. Furthermore, the output fna file was converted to Fastq by standalone phred33 conversion tool and submitted to the NCBI Biosample with accession no. SAMN02486448. The total six phyla containing 29 fungal species were identified [6]. Barcode sequence and Linker primer sequence are AAAAAAAC and TGGAGGGCAAGTCTGGTG respectively, which will be helpful for the advanced analysis.

## Figures and Tables

**Table 1:** Statistics of metagenomic data.

No.	Data description	Result
1	Total amplified sequences	8092
2	Base pairs count	2,947,772 bp
3	Sequence length	150–507 bp
4	Average sequence length	364.28 bp
5	GC content	45.6%
6	Average GC percent	45.9
7	Phred Quality Score	8–40
